# Self-esteem, social support and coping strategies of left-behind children in rural China, and the intermediary role of subjective support:a cross-sectional survey

**DOI:** 10.1186/s12888-021-03160-y

**Published:** 2021-03-17

**Authors:** Shu Cui, Fangshuo Cheng, Ling Zhang, Chao Zhang, Qiuyu Yuan, Cui Huang, Kai Zhang, Xiaoqin Zhou

**Affiliations:** 1 Chaohu Hospital, Anhui Medical University, Hefei, 238000 China; 2 Anhui Psychiatric Center, Anhui Medical University, Hefei, 238000 China; 3 The Third People’s Hospital of Fuyang, Fuyang, 236000 China; 4 Fuyang People’s Hospital, Fuyang, 236000 China

**Keywords:** Left-behind children, Coping strategies, Self-esteem, Social support

## Abstract

**Background:**

Negative coping strategies and behavioral problems are common among Chinese left-behind children, which are relate to a variety of negative consequences. At this stage of development, the relevant factors of coping strategies need to be further studied, in which social support and self-esteem are worthy of our attention. The aim of this study is to detect the current situation of self-esteem, social support, and coping styles of left-behind children (LBC) in rural China.

**Methods:**

322 children from 3 schools in China enrolled in this study, including 236 LBC and 86 non-left-behind children (NLBC) to assess self-esteem, social support and coping strategies.

**Results:**

The LBC group had lower self-esteem score and lower total social support (subjective support, objective support and support-seeking behavior) than the NLBC group. In terms of coping strategies, the LBC group was lower than the NLBC group in problem-solving and rationalization. The self-esteem score in LBC was significant positive associated with the subjective support score, objective support score, problem-solving and help-seeking score. In addition, self-esteem has significant mediating effect between subjective support and problem-solving, subjective support and help-seeking, respectively.

**Conclusions:**

The finding indicate that Chinese LBC’s self-esteem and social support need to be improved. Given the significant correlativity between self-esteem, subjective support and coping strategy, it is necessary to promote Chinese LBC’s self-esteem and social support, especially subjective support.

## Introduction

Since the 1980s, China has experienced rapid economic development. However, regions vary in terms of the rate of development, resulting in significant disparity in regional economies. An increasing number of adults from economically underdeveloped rural areas are moving to economically developed cities in search of better job opportunities. Due to the high cost of living and education in urban areas, most rural migrant residents cannot afford the education and daily living costs of their children, resulting in their children remaining in their rural hometowns. These children are known as left-behind children (LBC) [1–3]. LBC are children under 18 who were left behind in their rural communities, while one or both of their parents migrated into cities for work, and who had not lived with parents for > 6 months [4, 5]. According to the report of the All-China Women’s Federation in 2013, China had approximately 61.03 million LBC, which represented an increase of 2.42 million since 2005, accounting for 21.88% of the Chinese children population today [6]. Most reside in the rural areas of the following provinces: Sichuan Province, Guangdong Province, Jiangxi Province, Anhui Province, Henan Province, and Hunan Province [7].

According to previous studies in China and abroad, children who are left behind encounter a range of problems. LBC tend to display more emotional, behavioral, and learning problems, such as depression, non-suicidal self-injury, and game addiction [8–10].

There are many kinds of coping strategies: problem-solving, rationalization, help-seeking, fantasy, avoidance, and self-accusation. Generally speaking, coping strategies can be sorted into two types: immature and mature, among which immature coping strategies include avoidance, fantasy, and self-accusation. Mature coping strategies include problem-solving, help-seeking, and rationalization. These coping strategies can be used by all children, but when faced with difficulties, each child has their own unique set of coping strategies to which they are accustomed. In theory, the use of different coping strategies affects the final results of life events and then brings different psychological and emotional experiences to individuals [11, 12]. Previous studies indicate that, compared with non-left behind children (NLBC), LBC are less likely to use positive coping styles such as problem-solving [13]. If a child demonstrates social problem-solving deficits, or difficulties identifying problems and generating appropriate solutions, he will experience more hopelessness, depression, and suicide-related behaviors [14].

There are many definitions of social support. One definition is “a social network’s provision of psychological and material resources intended to benefit an individual’s capacity to cope with stress” [15] and another is “having or perceiving to have close others who can provide help or care, particularly during times of stress” [16]. Social support seems to be related to a range of psychological and behavioral mechanisms, including increased self-esteem and the use of active coping strategies [17].

Although the prediction mechanism of social support on coping strategies is not very clear, we think that self-esteem may have an intermediary effect on social support and mature coping strategies. Self-esteem describes how people evaluate themselves and the extent to which they accept themselves, resulting in a basic sense of self-worth [18]. Theoretically, a person’s environment can affect self-esteem, support from others, and their positive evaluation can improve self-esteem [19] and may increase individuals’ confidence in their abilities and efforts, and provide them with more resources to deal with difficulties in life. Previous studies have also revealed that social support can enhance a sense of self-worth, which in turn assists in maintaining or increasing reduced self-esteem in the face of adverse events [20]. In addition, self-esteem can provide individuals with a good sense of self-efficacy and further provide confidence when dealing with difficulties and adversity [21], thus enabling them to employ more mature coping strategies to face challenges [22].

Groups vary in coping styles and social support, and these differences are affected by individual characteristics and external environmental factors [23]. Previous literature indicates that LBC are characterized by an unwillingness to express their troubles to their guardians; their social support mainly comes from peers, incomplete parent-child relations, and limited and autonomous social interaction [24, 25]. However, our understanding of the subtle, indirect and complex relationship between individual characteristics and external environmental factors and coping styles is still incomplete.

In view of this background, we hypothesized that the self-esteem, social support, and coping styles of LBC would vary from those of NLBC and that self-esteem may be an important mediating variable between social support and coping strategies. This study aimed to address the questions: what is the current situation of self-esteem, social support, and coping styles of LBC, and what is the relationship between the three? This study assessed Chinese LBC as research participants to explore the relationship between self-esteem, social support, and coping styles, and to identify and describe the influence of social support and self-esteem on coping strategies. The conclusions of this study can be used to formulate and propose behavioral intervention strategies for LBC.

## Methods

### Participants

We conducted a cross-sectional survey in Anhui Province from January to March 2019. We selected schools and participants through cluster sampling. In the first step, we randomly selected three cities (Maanshan City, Bozhou City, and Chaohu City) from all cities in Anhui Province. In the second step, we randomly selected a rural middle school from each city. In the third step, we randomly selected two classes from each middle school and investigated all the students in these classes. A total of 350 students were recruited from six classes.

A total of 350 questionnaires were distributed, and questionnaires with missing values more than 5% were eliminated), or containing obviously false responses, 28 (8%) participants were excluded from analyses. As a result, data from 322 participants were analyzed. We used the most widely accepted definition of LBC in China: children or adolescents under 18 years old who remained in their home region while one or both of the parents migrated to other cities for work, and the separation exceeded six consecutive months in the past year. It has been suggested that a child cannot fully understand the questionnaire until the age of 14, so we included only LBC who were aged between 14 and 17 years. LBC who met any of the following conditions were further excluded: 1) physical illness, or inability to complete the survey; 2) auditory dysfunction or language disorder; 3) unconscious or delirious, and unable to clearly express oneself.

Prior to the survey, informed written consent was obtained from the parents, or guardians on behalf of the participating minors (< 16 years). In addition, verbal consent was obtained from each participant.

### Measures

All the interviewers were pre-trained graduate students majoring in clinical medicine. A self-designed questionnaire was used to collect demographic data of all participants, such as age, gender, number of siblings, parental marital status, parental education, and attachment type. Permissions were obtained from the relevant institutions to utilize all questionnaires (Rosenberg Self-esteem scale, Social support rating scale, Coping style questionnaire).

#### Self-esteem

In this study, the Chinese version of the Rosenberg Self-esteem Scale [18](SES) was used to evaluate the participants’ self-esteem. The scale consists of 10 items on a 4-point scale, ranging from 1 to 4. The total score was between 10 and 40. A higher score on the self-esteem scale indicates higher self-esteem. Those with scores ≤25, 26–32, and ≥ 33 were considered to have low self-esteem, moderate self-esteem, and high self-esteem, respectively. The Chinese version of the Rosenberg Self-esteem Scale has shown good reliability and validity for measuring self-esteem [26] and Cronbach’s α coefficient in this study was 0.87.

#### Social support

The Chinese version of the Social Support Rating Scale (SSRS) was used to evaluate participants’ social support [27]. The scale includes 10 items in three following dimensions: objective support, subjective support, and support-seeking behavior. The total score for social support is the sum of the 10 items. A higher score indicates a higher level of social support. The application of SSRS for Chinese children and adolescents has been confirmed in terms of its reliability and validity [12, 28] and in this study, the Cronbach’s α coefficient was 0.82.

#### Coping strategy

The Chinese version of the Coping Style Questionnaire (CSQ) was compiled by Xiao et al. [29] and accords with the behavior habits of the Chinese people. The scale consists of 62 items, germane to Chinese characteristics and Chinese coping habits. Items are rated as 1 (agree) or 0 (disagree). The questionnaire comprises six subscales, including both immature and mature coping strategies. Each subscale examines two dimensions, tendency, and effectiveness, and the score of each coping strategy is the sum of the score of tendency and effectiveness. Immature coping strategies include avoidance, fantasy, and self-accusation. Mature coping strategies include problem-solving, help-seeking, and rationalization. The CSQ is a scale widely used among Chinese adolescents and demonstrates good reliability and validity [30] and in this study Cronbach’s α coefficient was 0.81.

### Statistical analysis

The data are expressed as mean ± standard deviation. Group differences in demographic and other characteristics between the LBC and NLBC groups were compared using independent t-tests for continuous variables and chi-squared test for categorical variables. In addition, we used Spearman correlation coefficients to examine the correlation between self-esteem, social support, and coping strategy. Then, numerical variables in the LBC group were normalized to Z scores, and PROCESS V3.3 was used for mediation analysis [31]. In order to examine the explanatory mechanism of the significant relationship between subjective support and problem-solving ability, as well as subjective support and help-seeking ability, we tested the role of self-esteem as an intermediary variable in the LBC group. In the intermediary analysis, the bootstrap program was repeated 5000 times to verify the mediating effect of the above variables, and the confidence interval (CI) was 95%. When CI did not contain 0, the indirect effect was considered significant. All data were analyzed using SPSS 20.0. A two-tailed *p*-value of < 0.05 was considered statistically significant.

## Results

### Demographic data and characteristics of LBC and NLBC group

A total of 322 LBC cases were enrolled in our study, including 236 LBC and 86 NLBC cases. Table 1 shows the demographic data for all participants. No significant between-groups differences were observed for gender, age, and number of siblings (all *p* values > 0.05). However, there were significant differences in parental marriage, parental education, and child attachment type (all *p*-values < 0.01) (Table 1). Compared with NLBC, fewer LBC had a secure attachment type (Table 1). Parents of LBC had a higher rate of divorce and a lower level of education compared to parents of NLBC (Table 1).

**Table 1:** Demographic data and characteristics of LBC group and NLBC group

Variable		LBC group (*n* = 236)	NLBC group (*n* = 86)	t or χ^2^	p
Age (years)		14.41 ± 0.65	14.47 ± 0.68	−0.29	0.77
Gender	Male n (%)	125 (53%)	43 (50%)	0.22	0.64
	Female n (%)	111 (47%)	43 (50%)		
Number of siblings	No sibling n (%)	64 (27%)	23 (27%)	< 0.001	0.95
	At least one n (%)	172 (73%)	63 (73%)		
Parental marital status	Divorce n (%)	34 (14%)	3 (3%)	7.39	0.01
	Not divorced n (%)	202 (86%)	83 (97%)		
Parental education	Both are less than 9 years education n (%)	184 (78%)	54 (63%)	7.53	0.01
	One of them is more than 9 years education n (%)	52 (22%)	32 (37%)		
Attachment type	Secure n (%)	24 (10%)	27 (31%)	21.30	< 0.001
	Insecure n (%)	212 (90%)	59 (69%)		

LBC, left-behind children; NLBC, non-left-behind children.

### Comparison of self-esteem, social support, and coping strategy between LBC and NLBC group

As shown in Table 2, the LBC group had lower self-esteem scores than the NLBC group (*p* < 0.05). The LBC group was lower than the NLBC group in total social support, subjective support, objective support, and support-seeking behavior scores (all *p*-values < 0.05). In terms of coping strategies, the LBC group was lower than the NLBC group in problem-solving and rationalization, and the difference was significant. There was no significant difference between the two groups in other aspects of coping strategies, such as avoidance, self-accusation, and fantasy (all *p*-values > 0.05). However, after adjusting for the variables in Table 1 (age, gender, and attachment type of children, child number, marriage, and education of parents), only subjective support, objective support, and rationalization remained significantly different between groups.

**Table 2:** Comparison of self-esteem, social support, and coping strategy between the LBC and NLBC groups

Variables		NLBC group (n = 86)	LBC group (*n* = 236)	Before adjustment		After adjustment*	
				F	P	F	P
Self-esteem		30.19 ± 0.47	28.8 ± 0.28	6.35	0.012	1.63	0.294
	Low self-esteem (n)	12	50				
	Moderate self-esteem(n)	49	141				
	High self-esteem (n)	25	45				
Social support		36.55 ± 0.59	33.52 ± 0.36 -	19.52	< 0.001	9.43	0.002
	Subjective support	20.79 ± 3.23	19.29 ± 3.65	11.26	0.001	6.17	0.013
	Objective support	7.64 ± 1.77	6.93 ± 1.70	10.66	0.001	5.77	0.017
	Support-seeking behavior	8.12 ± 1.98	7.36 ± 2.01	8.99	0.003	2.48	0.116
Coping strategy	Problem-solving	2.14 ± 0.28	2.02 ± 0.39	6.65	0.01	2.43	0.120
	Help-seeking	1.94 ± 0.43	1.85 ± 0.52	2.01	0.157	0.34	0.562
	Rationalization	1.73 ± 0.40	1.61 ± 0.48	4.39	0.037	4.06	0.045
	Avoidance	1.64 ± 0.42	1.59 ± 0.55	0.44	0.509	0.04	0.834
	Self-accusation	1.42 ± 0.60	1.43 ± 0.60	0.04	0.84	0.01	0.987
	Fantasy	1.56 ± 0.49	1.58 ± 0.52	0.04	0.839	1.63	0.202

LBC, left-behind children; NLBC, non-left-behind children.

*Adjusted for age, gender, and attachment type of children, child number, marriage, and education of parents

### Correlation between self-esteem, social support, and coping strategy in LBC

The Spearman correlation analysis in Table 3 revealed that self-esteem was significantly positively correlated with subjective support, objective support, problem-solving, and help-seeking. In addition, subjective support was positively correlated with self-esteem and problem-solving, subjective support was positively correlated with self-esteem and help-seeking.

**Table 3:** Correlations among self-esteem, social support, and coping strategy in LBC

		1. Self-esteem	2. Subjective support	3. Objective support	4. Problem-solving	5. Rationalization	6. Self-accusation	7. Help-seeking	8. Fantasy	9. Avoidance
1	r	1.000	0.319^**^	0.198^**^	0.347^**^	−0.047	− 0.115	0.194^**^	− 0.006	− 0.086
	P		< 0.001	0.002	< 0.001	0.471	0.077	0.003	0.930	0.189
2	r		1.000	0.163^*^	0.382^**^	0.036	−0.010	0.159^*^	0.073	0.108
	P			0.012	< 0.001	0.579	0.874	0.014	0.265	0.099
3	r			1.000	0.142^*^	0.021	−0.071	0.106	0.022	−0.077
	P				0.030	0.743	0.276	0.105	0.741	0.236
4	r				1.000	0.357^**^	0.225^**^	0.421^**^	0.145^*^	0.245^**^
	P					< 0.001	< 0.001	< 0.001	0.026	< 0.001
5	r					1.000	0.513^**^	0.108	0.348^**^	0.429^**^
	P						< 0.001	0.097	< 0.001	< 0.001
6	r						1.000	−0.048	0.449^**^	0.501^**^
	P							0.463	< 0.001	< 0.001
7	r							1.000	−0.028	0.082
	P								0.673	0.210
8	r								1.000	0.500^**^
	P									< 0.001

### The mediating role of self-esteem between subjective support and problem-solving tendency and effectiveness

**Fig. 1:**
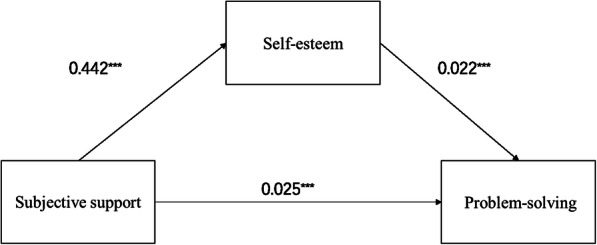
Model of Mediation Analyses. The arrow from Subjective support to Problem-solving represents the direct effect; the arrow from Subjective support to Problem-solving passing through Self-esteem, which is the mediator, represents the indirect effect. * *p* < 0.05; ** *p* < 0.01

Self-esteem can significantly mediate the relationship between subjective support and problem-solving. Evaluation of the overall and direct influence of subjective support on problem-solving ability indicated a significant effect of subjective support on problem-solving ability (all *p*-values < 0.001). Self-esteem was then introduced as an intermediary variable, and age, and gender as control variables into the regression equation, to calculate the indirect effect of the relationship between subjective support and problem-solving ability. The statistical significance in each path is shown in Table 4. Finally, Table 5 shows the bootstrap results of the mediating effect of self-esteem and its effect rate. The 95% CI value of the mediating effect of self-esteem did not include 0, indicating that there was a significant mediating effect, accounting for 27.1% of the total effect. Figure 1 shows an intermediary model in which self-esteem mediated subjective support and problem-solving.

**Table 4:** Regression analysis of the relationship between variables in the model

	Self-esteem			Problem-solving			Problem-solving		
	B	t	P	B	t	P	B	t	P
Subjective support	0.442	6.906	< 0.001	0.025	4.513	< 0.001	0.035	6.446	< 0.001
Gender	0.082	0.178	0.859	0.066	1.738	0.083	0.068	1.729	0.085
Age	−0.515	−1.476	0.141	−0.037	−1.286	0.199	−0.048	− 1.626	0.105
Self-esteem				0.022	4.690	0			
R^2^	0.134			0.181			0.124		
F	16.410			17.485			14.992		

Independent variable: subjective support; Dependent variable: problem-solving; Mediators: self-esteem.

**Table 5:** Bootstrap results for the mediating effect of self-esteem between subjective support and problem-solving ability

Effect type	Effect	BootSE	Bootstrap 95% CI LLCI	Bootstrap 95% CI ULCI	Effect ratio
Indirect effect	0.010	0.003	0.005	0.016	27.1%
Direct effect	0.025	0.006	0.015	0.036	72.6%
Total effect	0.035	0.005	0.025	0.045

### The mediating role of self-esteem between subjective support and help-seeking tendency and effectiveness

**Fig. 2:**
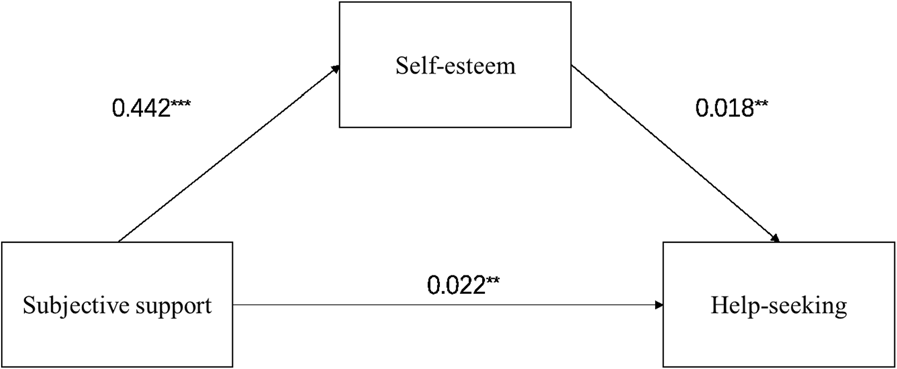
Model of Mediation Analyses. The arrow from Subjective support to Help-seeking represents the direct effect; the arrow from Subjective support to Help-seeking passing through Self-esteem, which is the mediator, represents the indirect effect. * *p* < 0.05; ** *p* < 0.01

Self-esteem can significantly mediate the relationship between subjective support and help-seeking. Evaluation of the overall and direct influence of subjective support on help-seeking ability indicated a significant effect of subjective support on help-seeking ability (all *p*-values < 0.001). We then introduced self-esteem as an intermediary variable, age, and gender as control variables into the regression equation, and calculated the indirect effect of the relationship between subjective support and help-seeking ability. The statistical significance in each path is shown in Table 6. Finally, Table 7 shows the bootstrap results of the mediating effect of self-esteem and its effect rate. The 95%CI value of the mediating effect of self-esteem did not include 0, indicating that there was a significant mediating effect, accounting for 26.1% of the total effect. Figure 2 shows an intermediary model in which self-esteem mediates subjective support and help-seeking.

**Table 6:** Regression analysis of the relationship between variables in the model

	Self-esteem			Help-seeking			Help-seeking		
	B	t	P	B	t	P	B	t	P
Subjective support	0.442	6.906	< 0.001	0.022	2.729	0.007	0.030	3.920	< 0.001
Gender	0.082	0.178	0.859	−0.032	−0.590	0.556	−0.031	− 0.558	0.577
Age	−0.515	−1.476	0.141	−0.041	−0.981	0.327	−0.050	−1.193	0.234
Self-esteem				0.018	2.660	0.008			
R^2^	0.134			0.072			0.051		
F	16.410			6.134			5.710		

Independent variable: subjective support; Dependent variable: problem-seeking; Mediators: self-esteem.

**Table 7:** Bootstrap results for the mediating effect of self-esteem between subjective support and help-seeking ability

Effect type	Effect	BootSE	Bootstrap 95% CI LLCI	Bootstrap 95% CI ULCI	Effect ratio
Indirect effect	0.008	0.004	0.001	0.016	26.1%
Direct effect	0.022	0.009	0.005	0.039	73.9%
Total effect	0.030	0.008	0.013	0.046

### Gender differences in self-esteem, social support, and coping strategies of LBC

Table 8 summarizes the differences in self-esteem, social support, and coping strategies between male LBC (*n* = 125) and female LBC (*n* = 111). No significant difference was observed between the groups in.

**Table 8:** Gender differences in self-esteem, social support, and coping strategies of LBC

Variables	Male(*n* = 125)	Female(*n* = 111)	t	P
Self-esteem	28.69 ± 4.43	28.94 ± 4.45	−0.43	0.668
Social support	33.55 ± 5.57	33.48 ± 5.37	0.10	0.917
Subjective support	19.21 ± 3.68	19.39 ± 3.64	−0.38	0.707
Objective support	7.10 ± 1.95	6.74 ± 1.36	1.69	0.093
Support-seeking behavior	7.39 ± 2.00	7.32 ± 2.03	0.26	0.797
Coping strategy				
Problem-solving	2.05 ± 0.38	1.99 ± 0.41	1.24	0.218
Help-seeking	1.82 ± 0.57	1.88 ± 0.46	−0.85	0.394
Rationalization	1.66 ± 0.50	1.55 ± 0.44	1.84	0.068
Avoidance	1.67 ± 0.54	1.51 ± 0.55	2.20	0.029
Self-accusation	1.49 ± 0.62	1.37 ± 0.58	1.58	0.116
Fantasy	1.65 ± 0.54	1.49 ± 0.48	2.47	0.014

Terms of self-esteem, social support, problem-solving, help-seeking, rationalization, and self-accusation (all *p* > 0.05). However, significant differences were observed between groups in avoidance and fantasy (all p < < 0.05).

## Discussion

The first purpose of this study was to determine the differences between LBC and NLBC in self-esteem, social support, and coping strategies. We observed that compared with the NLBC group, the LBC group had lower self-esteem and social support. Few LBC used mature coping strategies, such as problem-solving and rationalization.

Previous studies have shown that social support is one of the main protective factors to enhance the quality of parenting, children’s resilience and family happiness [32] . Gao and colleagues observed that the more contact there is between parents and child, the more social support and interpersonal relationships the left-behind children could achieve at school [28]. However, because the parents of LBC migrate to large cities for improved employment opportunities, they have little contact with their children. Therefore, according to our results, it can be inferred that the LBC group had lower social support than the NLBC group. Interestingly, our analyses revealed a positive correlation between self-esteem and subjective support among LBC. High self-esteem LBC had more subjective feelings of support in this study. Dai and colleagues reported that Western Chinese LBC display lower levels of happiness and self-esteem than NLBC [33]. They examined self-esteem among LBC in Sichuan Province in China using the Modified Harter Self-esteem Scale. Although they used different assessment scales than the current study, the results were similar. Low self-esteem was associated with depression, anxiety, internet addiction, and psychological and behavioral problems [7, 30, 34].

The second purpose of this study was to determine the relationship between self-esteem, social support, and coping strategies among the LBC group. Our results confirmed a positive relationship between self-esteem, social support, and mature coping strategies. Specifically, self-esteem was significantly positively correlated with subjective support, objective support, problem-solving, and help-seeking. These results indicate that self-esteem and social support are related to coping styles adopted by adolescents in that, adolescents with higher social support and self-esteem are more likely to adopt help-seeking and problem-solving behaviors. Our results are consistent with some previous literature in that positive problem orientation is significantly positively correlated with the two dimensions of self-esteem (self-competence and self-liking) among patients with anorexia [35]. A total of 92% of teenagers reported that social stigma and embarrassment prevented them from seeking professional help for mental health issues [36].

Our findings also indicate that when controlling for age and gender as covariables, self-esteem is not only the intermediary variable between subjective support and problem-solving ability, but also the intermediary variable between subjective support and help-seeking ability. Increased subjective social support may assist children to develop higher self-esteem, be more willing to solve problems in the face of difficulties, and more likely to solve problems successfully. In the process of dealing with problems, they may be more willing to turn to the people around them for help. On the contrary, a lack of subjective social support may be related to LBC’s negative attitude toward themselves. They may have the psychological characteristics of low self-esteem. People with low self-esteem are reluctant to accept themselves and often demean themselves, as expressed by feelings such as “I can’t do simple things”, “I am worthless”, lack of confidence in completing work, or solving problems. Additionally, they are often lonely and sensitive, ignore constructive criticism from their friends, are unwilling to open their hearts to others, and believe that asking for help is an acknowledgment of their inability, or fear that others will refuse to help. Therefore, it is possible that the LBC who lack subjective support are unwilling to solve the problem and seek help when facing difficulties.

In summary, our study emphasizes the influence of subjective support on the use of problem-solving and help-seeking coping strategies among LBC. Future studies should focus on social work in terms of providing more social support to LBC, including high-quality services among high-risk young people to improve parent-child communication, psychological counseling participation by teachers, engaging or educational extracurricular activities, and so on.

Our study has several limitations. First, is the cross-sectional survey design, thus we cannot interpret the cause-effect relationship among self-esteem, social support, and coping strategy. Second, the participants were selected from three cities in Anhui Province. Although our study adopted the method of cluster sampling, the results should be generalized to all LBC in China with caution. We chose teenagers over the age of 14 because it was assumed that younger children would be unable to accurately understand the meaning of the questions on the scales we used. The results among 14-to 17-year-olds may not be universal to the entire population of LBC.

## Conclusions

Compared with NLBC, the LBC group had lower levels of self-esteem and social support. Among the LBC group, self-esteem was significantly positively correlated with subjective support, objective support, problem-solving, and help-seeking. Self-esteem played an intermediary role in the relationship between adolescents’ subjective support and problem-solving or help-seeking tendencies and effectiveness. It is hoped that these findings will have some implications on how to improve LBC coping strategies (problem-solving and help-seeking ability).

## Data Availability

All the data supporting our findings have been presented in the manuscript; the datasets used and/or analyzed during the current study are available from the corresponding author on reasonable request.

## Abbreviations

LBC: left-behind children
NLBC: non-left-behind children
SES: Rosenberg Self-esteem Scale
SSRS: Social Support Rating Scale
CSQ: Coping Style Questionnaire
